# Performance analysis of two typical greenhouse lettuce production systems: commercial hydroponic production and traditional soil cultivation

**DOI:** 10.3389/fpls.2023.1165856

**Published:** 2023-07-04

**Authors:** Lichun Wang, Songrui Ning, Wengang Zheng, Jingyu Guo, Youli Li, Yinkun Li, Xiaoli Chen, Alon Ben-Gal, Xiaoming Wei

**Affiliations:** ^1^ Intelligent Equipment Research Center, Beijing Academy of Agriculture and Forestry Sciences, Beijing, China; ^2^ Key Laboratory of Eco-hydraulics in Northwest Arid Region of China, Xi’an University of Technology, Xi’an, China; ^3^ College of Science, Edith Cowan University, Perth, Western Australia, Australia; ^4^ Soil, Water and Environmental Sciences, Agricultural Research Organization – Volcani Institute, Gilat Research Center, Mobile Post, Negev, Israel

**Keywords:** buffer capacity, economic benefit, nitrate, quality, vegetable production, water productivity

## Abstract

**Introduction:**

Due to the shortage of land and water resource, optimization of systems for production in commercial greenhouses is essential for sustainable vegetable supply. The performance of lettuce productivity and the economic benefit in greenhouses using a soil-based system (SBS) and a hydroponic production system (HPS) were compared in this study.

**Methods:**

Experiments were conducted in two identical greenhouses over two growth cycles (G1 and G2). Three treatments of irrigation volumes (S1, S2, and S3) were evaluated for SBS while three treatments of nutrient solution concentration (H1, H2, and H3) were evaluated for HPS; the optimal levels from each system were then compared.

**Results and discussion:**

HPS was more sensitive to the effects of environmental temperature than SBS because of higher soil buffer capacity. Compared with SBS, higher yield (more than 134%) and higher water productivity (more than 50%) were observed in HPS. We detected significant increases in ascorbic acid by 28.31% and 16.67% and in soluble sugar by 57.84% and 32.23% during G1 and G2, respectively, compared with SBS. However, nitrate accumulated in HPS-grown lettuce. When the nutrient solution was replaced with fresh water 3 days before harvest, the excess nitrate content of harvested lettuce in HPS was removed. The initial investment and total operating cost in HPS were 21.76 times and 47.09% higher than those in SBS, respectively. Consideration of agronomic, quality, and economic indicators showed an overall optimal performance of the H2 treatment. These findings indicated that, in spite of its higher initial investment and requirement of advanced technology and management, HPS was more profitable than SBS for commercial lettuce production.

## 1 Introduction

In light of rapid urbanization and population growth combined with limited water and land resources, expanding large cities are challenged to attain the necessary fresh vegetable supply for citizens from traditional agricultural production in their hinterlands (Orsini et al., 2013; Thomaier et al., 2015). Therefore, the state of food security in large cities is fragile, especially when the agri-fresh food supply chain is disturbed, as occurs periodically because of environmental or anthropogenic disruptions. Owing to the shortage of farmable land and high labor costs for vegetable production, planners tend to pursue intensive agricultural production systems that can provide high yields and economic benefits (Battersby and Marshak, 2013; Amos et al., 2018).

To overcome the shortage of farmland, soilless culture technologies are widely used (Goodman and Minner, 2019). There are various forms of soilless culture that have been used including hydroponics, aeroponics, and culture in various soilless media. These systems have additional benefits of reduced crop yield loss caused by soil-borne pests and by soil salinity accumulation (Hogan et al., 2006; Savvas and Gruda, 2018). Professional hydroponic production systems (HPSs) are attractive because of the absence of solid waste and the highly efficient energy use. HPS is considered among the most sustainable of agricultural technologies (Urrestarazu, 2013). Numerous studies confirmed that hydroponics can produce efficient nutrient utilization, higher-density planting, and increased yield per area compared with traditional soil-based systems (SBS) (Sardare and Admane, 2013). In a simple comparison of HPS with SBS, Majid et al. (2021) found that hydroponic systems produced 40% larger lettuce plants. Compared with SBS, Leal et al. (2020) observed that HPS could mitigate the influence of salinity stress and obtain higher yield when using water containing high concentrations of salt. Compared with simple HPS, large commercial scale HPS is more practicable and applicable for fresh vegetable production (Quagrainie et al., 2018). However, few studies have compared HPS to SBS regarding leafy vegetable yield per unit area in an urban setting.

The improvement of living standards is accompanied by increased awareness of the importance of high-quality vegetables in human diets (Fanasca et al., 2006; Ferguson et al., 2014). In HPS, the nutrient supply can be controlled precisely, therefore promoting high product quality. Buchanan and Omaye (2013) observed that ascorbic acid content of hydroponically grown lettuce was more than 90% higher than that in SBS. Verdoliva et al. (2021) found that tomatoes grown in deep water culture HPS had higher beta-carotene and lycopene contents than under soil cultivation. Majid et al. (2021) reported that HPS produced lettuce with higher total soluble solids, protein, and crude fiber content than the SBS. However, many researchers have pointed out that the nitrate contents of hydroponic leaf greens can be much higher than in SBS (Bian et al., 2016; Pace et al., 2018), leading to a latent threat to the health of consumers (Lei et al., 2018; Rocha et al., 2020). Thus, reducing the nitrate content in hydroponic crops is of widespread concern for researchers, growers, and consumers (Bian et al., 2016).

HPS has been substantiated as an environmentally friendly technique for agricultural production, much due to the ability to minimize waste solution drainage and reduce fertilizer needs and costs (Grewal et al., 2011). However, some growers often criticize such production systems due to their high installation costs including the nutrient solution tank, cycling pump, and growth system (Barbosa et al., 2015; Putra and Yuliando, 2015). Profit is vital to both prosperity of individual farmers and national trade balance, especially in the developing countries. Therefore, the economic feasibility of commercial HPS compared with traditional SBS should be evaluated.

Crop growth and yields and benefits are influenced by supplement of fertilizer (concentration of nutrient solution) and water (irrigation) in HPS and SBS, respectively. The aims of this study were to systematically assess the relative impact of nutrient solution concentrations and irrigation water levels on plant growth between HPS and SBS grown under conditions of large-scale commercial greenhouses. In the study, yield, nutritional quality, production efficiency, and crop root zone environmental variables were measured and compared for the two cultivation systems. The findings are expected to promote the adoption and development of hydroponics systems.

## 2 Materials and methods

### 2.1 Experimental conditions

An experiment was conducted over two growth cycles at The Research Center of Intelligent Equipment for Agriculture, Beijing Academy of Agriculture and Forestry Sciences, Beijing, China (39.95° N, 116.28° E) from 3 March to 6 May 2021. This area has a sub-humid continental monsoon climate with an annual mean temperature of 12.5°C.

The experimental greenhouses were each constructed of a steel frame structure (11.8 m × 28 m), and covered with glass. Two identical side-by-side greenhouses were used for HPS and SBS, separately. Greenhouse inside air temperature was controlled using fan-pad and cooling systems. Lettuce seeds (*Lactuca sativa* L. cv. Flandria, Rijk Zwaan seed, Holland) were sown in 72-cell plug trays in a growth chamber. Air temperature, CO_2_ level, and relative humidity (RH) in the growth chamber were maintained at 22/18°C (day/night), 350 μmol mol^−1^, and 65%, respectively. The seedlings were irrigated with half-strength Hoagland nutrient solution. The lettuce seedlings were transplanted in the greenhouses on 3 March 2021 for the first growth cycle (G1) and 8 April 2021 for the second cycle (G2). During the whole experimental periods, the maximum and minimum temperature, RH, and solar radiation were recorded by a weather station (Campbell Scientific, Inc., USA) installed in the greenhouse.

### 2.2 Treatment design

#### 2.2.1 Soil-based system

The tillage layer (0–30 cm) of soil in the SBS greenhouse had a bulk density of 1.32 g cm^−3^, a field capacity water content of 0.26 cm^3^ cm^−3^, and a soil water electrical conductivity (EC_1:5_, 1:5 soil/water ratio) of 220 μS cm^−1^. The soil texture is classified as sandy loam (USDA). The soil available contents of nitrogen (N), phosphorus (P), and potassium (K) were 143, 93, and 150 mg kg^−1^.

Before transplanting, organic fertilizer with 6,000 kg ha^−1^ (i.e., the contents of N, P_2_O_5_, and K_2_O were 7.14, 0.06, and 3.68 g kg^−1^, respectively) was applied to the soil surface and the root zone soil was turned over to a depth of 30 cm. 75 kg ha^−1^ of water-soluble compound fertilizer (3:1:1, N:P_2_O_5_:K_2_O) was applied at 22 and 19 days after transplanting (DAT) during G1 and G2, respectively.

The irrigation amount was determined according to pan evaporation. A standard and automatic water surface evaporator (20 cm diameter) was installed in the greenhouse to measure daily pan evaporation (*E_p_*). The lettuce was irrigated whenever the accumulated *E_p_* (*AE_p_*) reached 15 mm. Three treatments were set according to different irrigation volumes, i.e., 0.7*AE_p_* (S1), 0.9*AE_p_* (S2), and 1.1*AE_p_* (S3). Each treatment was repeated three times including one in a lysimeter enabling calculation of evapotranspiration. For each treatment, the plot area was 0.6 m ×1 m. The plots were arranged randomly. Lettuce was grown in the surrounding experimental plots to minimize edge effects. The lettuce seedlings were transplanted at a plant spacing of 20 cm × 20 cm, with a plant density of 17.5 plants m^−2^. Water was applied using a 5-L watering can to simulate sprinkling irrigation. Immediately following transplanting, 40 mm of water was applied to each plot. The total amounts of water applied to S1, S2, and S3 were 73, 94, and 116 mm during G1, and 113, 155, and 197 mm during G2, respectively.

#### 2.2.2 Hydroponic production system

**Figure 1:**
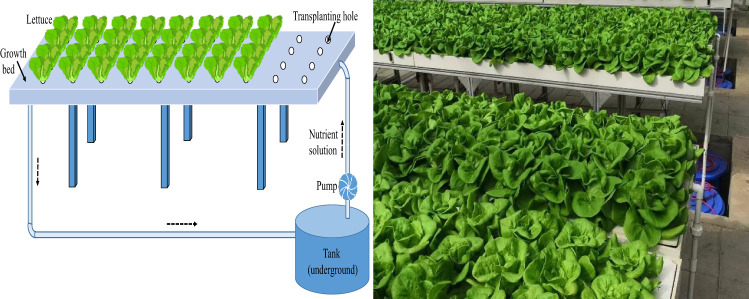
Isometric view of the hydroponic growth system.

Growth channels were installed in the greenhouse. The growth channels were made of polyvinyl chloride (PVC) with a size of 200 cm (length) × 60 cm (width) ×8 cm (height). Each channel was covered with a PVC board (199 cm × 59 cm) having 52 holes (3 cm in diameter) in the same row and column space of 15 cm, providing a plant density of 29.8 plants m^−2^ (**Figure 1**). A 60-L plastic tank was installed underground to supply nutrient solution. A circulation pump was installed in the tank to pump the nutrient solution to the growth channel. Excess nutrient solution was drained out of the growth channel back to the tank via an outlet pipe. The circulation pump operated from 7:00 a.m. to 6:00 p.m. every day.

Based on a modified half-strength “Hoagland nutrient solution” (HS), three different nutrient solution concentrations equal to 0.7 HS (H1), 1.0 HS (H2), and 1.3 HS (H3) were prepared and applied. Each treatment was replicated three times. The electrical conductivity of nutrient solution concentration (*ECw*) was 0.73, 0.96, and 1.28 dS m^−1 ^for treatments H1, H2, and H3, respectively. The pH of nutrient solutions was adjusted to a range from 5.5 to 6.5 with 10% H_2_SO_4_ or NaOH solution. The variations of solution *ECw* and pH are probably due to the selective ion uptake of plants.

Moreover, to reduce the nitrate content of lettuce before harvest, for the treatments H1, H2, and H3 during G2, one hydroponic growth channel was chosen to change the nutrient solution to 80 L of pure fresh water (FW) 3 days before harvest. These treatments were named FWH1, FWH2, and FWH3, respectively.

### 2.3 Measurements

In SBS, the soil temperature in the root zone was measured using a thermocouple installed at a depth of 15 cm from the soil surface in lysimeter plot of treatment S2 and connected to a data logger (Testo 176-T4, AG, Germany). Volumetric soil water content at 15 cm was measured using a sensor installed in each lysimeter (ECH_2_O EC-5 sensor, Decagon Devices Ltd, Pullman, USA). Data were stored on a data logger (EM50, Decagon Devices Ltd, Pullman, USA).

In each treatment of HPS, *ECw*, temperature, and water level were monitored using a sensor (CT-10 sensor, Decagon Devices Ltd, Pullman, USA) in the nutrient solution tank. Data were stored on a data logger (EM50, Decagon Devices Ltd, Pullman, USA). Whenever the volume of solution in the tank dropped to less than 5 L, 40 L of nutrient solution was added. The nutrient solution volume (*V*, L) was determined by its relationship with the water level (*H*, mm) in the tank: *V* = 0.12*H* − 0.93, *R* ^2^ = 0.95. The water consumption (evapotranspiration) for each treatment was calculated using the water balance method.

For each treatment, three lettuce shoots were sampled randomly every 7 days to determine fresh weight. The leaves were removed from each plant and leaf area analyzed using a leaf area meter (LI-COR Inc., Lincoln, Nebraska, USA). Yield was determined as the fresh weight of lettuce at the last sampling event and presented as mass per unit greenhouse area (kg m^−2^).

At harvest, the contents of soluble sugar, vitamin C, and nitrate (fresh weight basis) were determined with 2,6-dichloro-indophenol titration, anthrone ethyl acetate colorimetic method, and salicylic acid method (Li, 2000), respectively. The crude fiber was measured on a dry weight basis using acid digestion (Li, 2000).

Lettuce quality parameters (i.e., the contents of soluble sugar, vitamin C, nitrate, and crude fiber) for the three treatments where nutrient solution was replaced by fresh water prior to harvest under HPS were also measured in G2.

Water productivity (*WP*, kg m^−3^) was calculated by:


(1) $$WP=\frac{Y}{AWC}$$

where *Y* is yield expressed as fresh weigh of lettuce per unit area, kg m^−2^; and *AWC* is the accumulated water consumption of lettuce per m^2^ during the whole growth cycle, m^3^ m^−2^.

### 2.4 Economic performance

To evaluate the economic viability in HPS and SBS, modern finance theory approaches were employed. We began by estimating that the average size of a commercial solar greenhouse in China is typically over 800 m^2^ (Sun et al., 2019). We next aimed to supply a sample budget for growers based on 1,000 m^2^ of commercial greenhouse. For commercial vegetable production in SBS, it is not practical to manually use watering cans for irrigation. An irrigation pump and differential pressure tank were considered to apply water and fertilizer to crops. The economic impacts of the two culture systems were assessed by the net present value (*NPV*) and the benefit-to-cost ratio (*BCR*). As the experiments were conducted in two identical greenhouses, the cost of land and greenhouse construction were not considered since they were unnecessary for the comparison regarding economic feasibility of HPS and SBS. Data related to yield, fixed costs (e.g., growth channel, frame, cycle pump, etc.), and variable costs (e.g., fertilizer, seeds, electricity power, etc.) were considered. The price of harvested lettuce and labor costs were collected by a market survey. A 10-year economic life was assumed for the two growth systems, with the investment potentially generating benefits during this period (Sait and Ayse, 2009). According to the surveys, leafy lettuce was assumed to be harvested 10 and 8 times annually in HPS and SBS, respectively.

The *NPV* is the present value of future return and calculated as (Grafiadellis et al., 2000):


(2) $$NPV=\sum_{t=1}^{n}\frac{R_{t}}{{\left(1+i\right)}^{t}}-I_{0}$$

where *n* is the economic life in years; *t* is the production system’s duration in years; *R_t_* is the net return at year *t*, $; *i* is the discount rate, reflecting the financial market and product price equaling 8%; and *I*_0_ is the initial investment, $.

The *BCR* (the ratio of benefit to cost) was used to evaluate economic productivity of the two typical growth systems (Majid et al., 2021):


(3) $$BCR=\frac{\sum_{t=1}^{\text{n}}R_{t}{\left(1+i\right)}^{-t}}{\sum_{t=1}^{\text{n}}C_{t}{\left(1+i\right)}^{-t}}$$

where *C_t_* is the cost during period *t*, $.

Cost and benefit values were expressed in US dollars ($). The average exchange rate between the Chinese currency (RMB) and US dollars in 2020 was 6.90 RMB per US dollars (NBSC, 2021). The annual average price of lettuce in China was 4.00 RMB per kg ($0.58 per kg, Ministry of Agriculture and Rural Affairs of China, http://zdscxx.moa.gov.cn:8080/nyb/pc/search.jsp). The decision rule was: if *NPV* > 0 or *BCR* > 1, the project would be successful; otherwise, the project would be considered a failure (Grafiadellis et al., 2000).

### 2.5 Multicriteria assessment

For the multicriteria evaluation of lettuce production system performance under different irrigation levels and nutrient solution concentration treatments, a Delphi approach was used to select seven indicators to be used by the evaluation teams. The indicators included yield, water productivity, *BCR*, net return, ascorbic acid, soluble sugar, and nitrate content. The main criteria of agronomic and economic performance were transformed to a 0–10 scale using minimum values (Min) and maximum values (Max) of each indicator, with 10 being the best performance and 0 being the worst performance: Scale = [10 × (Value – Min)/(Max – Min)] for the yield, water productivity, ascorbic acid, soluble sugar, *BCR*, and net return, and Scale = 10 − [10 × (Value − Min)/(Max − Min)] for nitrate content (Pelzer et al., 2012). The indicators’ weights of yield, water productivity, ascorbic acid, soluble sugar, nitrate content, *BCR*, and net return were determined as 0.25, 0.08, 0.075, 0.045, 0.10, 0.15, and 0.30, respectively, by consulting with 11 senior experts. A radar map, considering the weighted indicators, was used to comprehensively evaluate the performance of the different treatments.

### 2.6 Data analysis

The one-way analysis of variance (ANOVA) was performed using the SPSS19.0 software (SPSS, USA). Treatment means were compared, and differences between means were conducted by Tukey’s multiple range test at the 5% significance level (*p* ≤ 0.05). The whole growth cycle of lettuce growth characteristics and yields at harvest of three irrigation water or nutrient solution concentration level treatments were analyzed to choose the optimal, and only then were HPS and SBS compared by independent-samples *t*-test. The lettuce quality characteristics were compared among different treatments in each growth cycle. Graphs were produced using MS Excel 2019 (Microsoft Co. WA, USA) and MATLAB2017a software (The MathWorks Co. USA).

## 3 Results

### 3.1 Meteorological conditions

**Figure 2:**
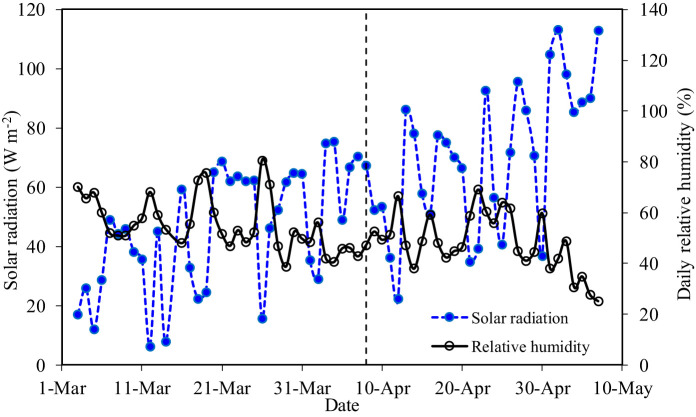
Daily solar radiation and relative humidity in the greenhouse.

Daily solar radiation and relative humidity (RH) in the greenhouse are shown in **Figure 2**. The variation of solar radiation during G2 was more stable than that during G1, which showed an increasing trend after 14 April. The highest solar radiation was 113.1 W m^−2^ on 2 May. The RH, which decreased with the prolongation of the growth period, was higher during G1 than during G2. The average solar radiation and RH were 44.52 W m^−2^ and 55.64% during G1, and 70.38 W m^−2^ and 48.07% during G2, respectively. In general, changes in RH were inverse to those of solar radiation.

**Figure 3:**
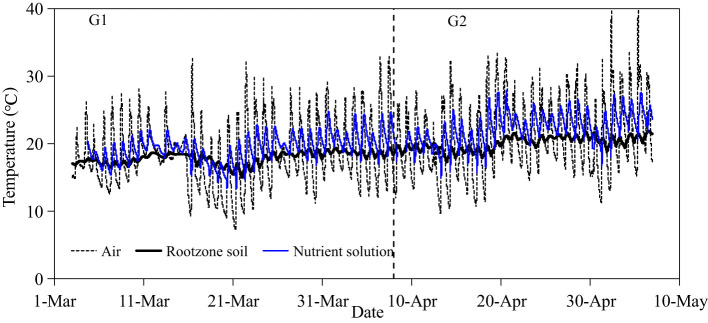
Temperature dynamics in greenhouse, nutrient solution, and soil.

**Figure 3** illustrates the temperature dynamics of greenhouse atmosphere, nutrient solution, and soil. The minimum and maximum air temperatures in the greenhouse were 7.3 and 32.6°C; during G1, and 9.7 and 40.4°C; during G2, respectively. The nutrient solution temperature in the underground tank fluctuated with the air temperature of greenhouse but at a lower amplitude. The temperature in the nutrient solution ranged from 13.3 to 24.8°C; during G1 and from 15.0 to 27.9°C; during G2. In the more buffered soil, root zone temperatures of the SBS were relatively stable ranging from 15.0 to 19.4°C; during G1 and from 17.3 to 21.9°C; during G2.

### 3.2 Soil water content

**Figure 4:**
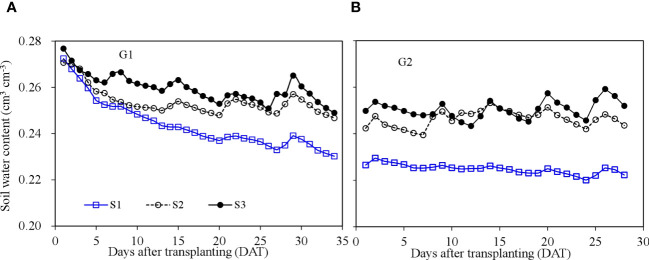
Soil water content dynamics in the soil-based system during the two growth cycles. G1 **(A)** the first cycle (3 March to 8 April 2021), G2 **(B)** the second cycle (9 April to 6 May 2021).

Root zone soil water content in SBS increased significantly after irrigation and gradually decreased between irrigation events (**Figure 4**). The initial soil moisture (close to 0.28 cm^3^ cm^−3^) of each treatment during G1 was high following pre-transplanting irrigation. The soil water content then decreased with time, especially from 1 to 7 DAT. Treatment S1, with the smallest irrigation volume, had the lowest soil water content, and treatment S3, with greater irrigation volume, had consistently the highest soil water content. The average root zone soil water content of treatments S1, S2, and S3 was 0.24, 0.25, and 0.26 cm^3^ cm^−3^ during G1, and 0.22, 0.24, and 0.25 cm^3^ cm^−3^ during G2, respectively.

### 3.3 Nutrient solution EC (*ECw*)

**Figure 5:**
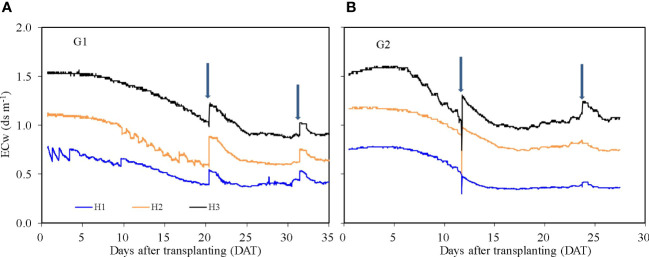
Nutrient solution electric conductivity (*ECw*) for treatments H1, H2, and H3 in the hydroponic production system during the two growth cycles. G1 **(A)** the first cycle (3 March to 8 April 2021), G2 **(B)** the second cycle (9 April to 6 May 2021). H1 (0.7 HS), H2 (1 HS), and H3 (1.3 HS); HS is half- strength Hoagland solution. Arrows represent times of replenishment of nutrient solution.

**Figure 6:**
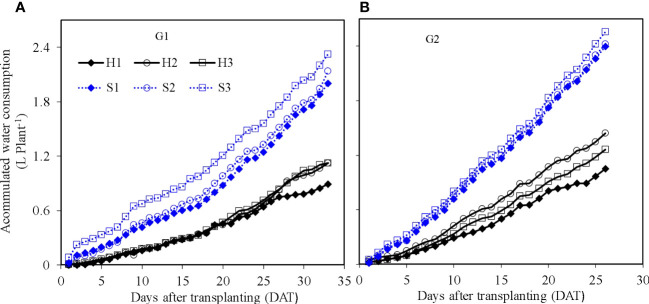
Accumulated evapotranspiration of treatments under hydroponic production system and soil-based system during two growth cycles (G1 and G2). G1 **(A)** the first cycle (3 March to 8 April 2021), G2 **(B)** the second cycle (9 April to 6 May 2021). S1 (0.7 *AE_p_*), S2 (0.9 *AE_p_*), and S3 (1.1 *AE_p_*); *AE_p_* is accumulated daily pan evaporation. H1 (0.7 HS), H2 (1 HS), and H3 (1.3 HS); HS is half-strength Hoagland solution.

The *ECw* reflects the concentration of nutrients in the HPS solution (**Figure 5**). During both G1 and G2, the EC of nutrient solution declined over time as water and solutes were taken up by roots. The *ECw* increased sharply with the addition of nutrient solution to the growth system. The average *ECw* for treatments H1, H2, and H3 was 0.52, 0.80, and 1.21 dS m^−1^ during G1 and 0.51, 0.94, and 1.22 dS m^−1^ during G2, respectively. *ECw* was observed to increase after 20 DAT from 0.98 to 1.07 dS m^−1^ for treatment H3 during G2. This slight rise may be due to enhanced transpiration and therefore process of concentration of the solution caused by higher temperatures during G2 (**Figure 6**).

### 3.4 Accumulated evapotranspiration

Plant-scale accumulated evapotranspiration increased over time together with plant growth (**Figure 6**). The lettuce evapotranspiration was influenced by the irrigation amount in SBS and nutrient solution concentration in HPS. Evapotranspiration accumulated per plant in SBS was higher than that in HPS. Taking the period of G1 at 25 DAT as an example, the per-plant accumulated evapotranspiration of S1, S2, and S3 was 1.24, 1.33, and 1.56 L in SBS, and that of H1, H2, and H3 was 0.64, 0.69, and 0.71 L in HPS, respectively. The highest per-plant accumulated evapotranspiration was observed under the same treatment S3 during both G1 and G2.

### 3.5 Growth characteristics

**Figure 7:**
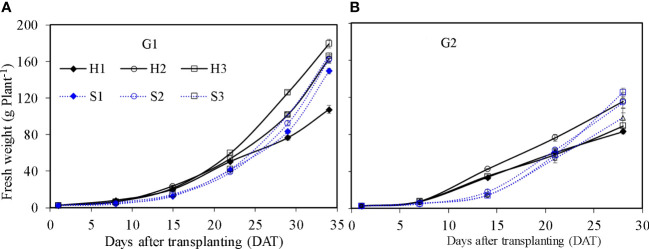
Fresh weight of lettuce per plant in the hydroponic production and soil-based systems. G1 **(A)** the first cycle (3 March to 8 April 2021), G2 **(B)** the second cycle (9 April to 6 May 2021). H1 (0.7 HS), H2 (1 HS), and H3 (1.3 HS); HS is half-strength Hoagland solution. S1 (0.7 *AE_p_*), S2 (0.9 *AE_p_*), and S3 (1.1 *AE_p_*); *AE_p_* is accumulated daily pan evaporation. Error bars represent standard error of three replications.

Shoot fresh weight of lettuce in SBS and HPS increased with time (**Figure 7**). The shoot fresh weight of lettuce was influenced significantly by irrigation level and growth season for SBS, and by nutrient solution concentration and growth season for HPS (*p* ≤ 0.05). At harvest, the maximum shoot fresh weight of lettuce for HPS was observed under treatments H3 during G1 (179.32 g plant^−1^) and H2 during G2 (115.61 g plant^−1^). The maximum shoot fresh weight of lettuce for SBS was observed under treatments S3 during G1 and G2 (166.30 and 125.36 g plant^−1^). For the optimal treatment of each system, lettuce shoot fresh weight in HPS was significantly higher than SBS during G1 (*p* ≤ 0.05). Difference in shoot fresh weight between the two systems was not obvious during G2 (*p* > 0.05). The dry weight of lettuce per plant under different treatments had similar trends to the fresh weight (data not shown).

**Figure 8:**
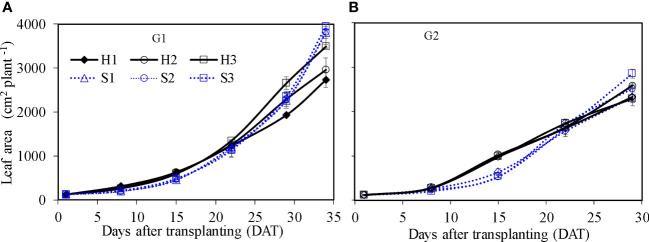
Leaf area of lettuce per plant in hydroponic production and soil-based systems. G1 **(A)** the first cycle (3 March to 8 April 2021), G2 **(B)** the second cycle (9 April to 6 May 2021). H1 (0.7 HS), H2 (1 HS), and H3 (1.3 HS); HS is half-strength Hoagland solution. S1 (0.7 *AE_p_*), S2 (0.9 *AE_p_*), and S3 (1.1 *AE_p_*); *AE_p_* is accumulated daily pan evaporation. Error bars represent standard error of three replications.

**Figure 9:**
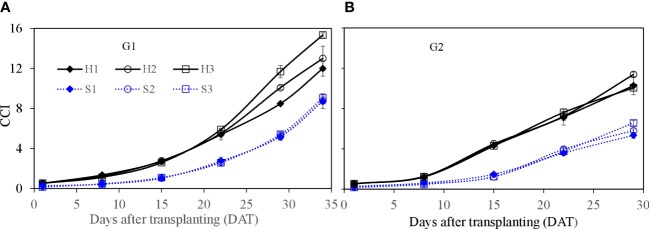
Canopy cover index (CCI) of lettuce per plant for hydroponic production and soil-based systems. G1 **(A)** the first cycle (3 March to 8 April 2021), G2 **(B)** the second cycle (Apr 9 to May 6, 2021). H1 (0.7 HS), H2 (1 HS), and H3 (1.3 HS); HS is half-strength Hoagland solution. S1 (0.7 *AE_p_*), S2 (0.9 *AE_p_*), and S3 (1.1 *AE_p_*); *AE_p_* is accumulated daily pan evaporation. Error bars represent standard error of three replications.

**Figure 10:**
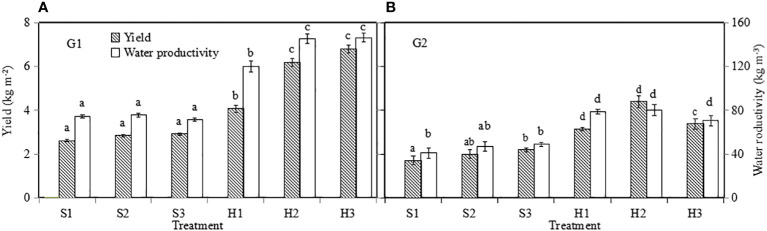
Yield presented as a function of greenhouse area (Y m^–2^) and water productivity of lettuce. G1 **(A)** the first cycle (3 March to 8 April 2021), G2 **(B)** the second cycle (9 April to 6 May 2021). H1 (0.7 HS), H2 (1 HS), and H3 (1.3 HS); HS is half-strength Hoagland solution. S1 (0.7 *AE_p_*), S2 (0.9 *AE_p_*), and S3 (1.1 *AE_p_*); *AE_p_* is accumulated daily pan evaporation. Error bars represent standard error of three replications. Different letters indicate significant difference at *p* ≤ 0.05 tested with one-way ANOVA.

Leaf area of single lettuce plants (**Figure 8**) showed similar growth dynamics as those found for fresh weight. Leaf area in HPS was significantly higher at the earlier growth stage (e.g., from 8 to 22 DAT) than that in SBS (*p* ≤ 0.05). After 22 DAT, the increase in leaf area in SBS was faster than that in HPS. At harvest, the leaf area in HPS was significantly lower than that in SBS (*p* ≤ 0.05). Considering the different plant density, the leaf growth was expressed as canopy cover index (CCI). Owing to the higher plant density, the CCI in HPS was higher than that in SBS (**Figures 9**, **10**). The nutrient solution concentration had a significant effect on the CCI of lettuce in HPS during the later period of experiment (*p* ≤ 0.05). In HPS, the highest CCI was observed under treatments H3 during G1 (15.34) and H2 during G2 (11.38). In SBS, the highest CCI was observed under treatment S3 during G1 (6.89) and G2 (6.57). The difference of CCI between HPS and SBS was more obvious than the leaf area.

### 3.6 Yield and *WP*

#### 3.6.1 Yield

Lettuce yield was influenced by growth season and irrigation level in SBS (*p* ≤ 0.05), and by growth season and nutrient solution level in HPS (*p* ≤ 0.05). For the optimal treatment of each system, lettuce yield (**Figure 10**) in HPS was significantly higher than that in SBS (*p* ≤ 0.05). During G1, the highest yield (6.81 kg m^−2^) was observed in HPS treatment H3, which was 134% higher than the highest yield (2.91 kg m^−2^) found in SBS treatment S3. During G2, the yield of lettuce for each treatment was lower than that during G1, probably due to its shorter growth period and plant stress due to higher greenhouse temperatures.

#### 3.6.2 WP

Similar to the lettuce yield, for the optimal treatment in each system, the *WP* (**Figure 10**) in HPS was 102.91% (during G1) and 50.2% (during G2) higher than SBS (*p* ≤ 0.05). The *WP* during G2 was lower than that during G1 under the same treatments. *WP* of H3 was 146.29 kg m^−3^ during G1, which was 107% higher than the 70.62 kg m^−3^ found during G2.

### 3.7 Product quality

In SBS, lettuce ascorbic acid, soluble sugar, crude fiber, and nitrate content were not influenced by the irrigation level (**Table 1**; *p* > 0.05). In HPS, the ascorbic acid content was not influenced by the nutrient solution concentration (**Table 1**; *p* > 0.05). The soluble sugar and crude fiber content under treatment H3 were significantly higher than H1 and H2 during G1 (**Table 1**). In HPS, lettuce nitrate content increased significantly as a function of increasing nutrient solution concentration (**Table 1**). For the optimal treatment of H2 in HPS, the ascorbic acid and soluble sugar content were 28.05% and 85.29% higher than the optimal treatment of S3 in SBS.

**Table 1:** Quality parameters, ascorbic acid, soluble sugar, nitrate content, and crude fiber of lettuce under different treatments during two growth cycles.

Growth cycle	Treatment	Ascorbic acid (mg kg^−1^)	Soluble sugar (mg kg^−1^)	Nitrate content (mg kg^−1^)	Crude fiber (%)
G1	S1	112.34 ± 5.59a	1.21 ± 0.09b	838.59 ± 10.41a	1.20 ± 0.04b
	S2	119.76 ± 2.82a	1.04 ± 0.04a	849.61 ± 20.36a	1.00 ± 0.08a
	S3	113.98 ± 2.54a	1.02 ± 0.12a	865.03 ± 4.83a	1.37 ± 0.09c
	H1	154.47 ± 5.34c	1.69 ± 0.11c	1,183.26 ± 39.73b	1.35 ± 0.16b
	H2	149.80 ± 5.52bc	1.89 ± 0.11c	1,428.85 ± 44.61c	1.52 ± 0.03bc
	H3	145.95 ± 2.13b	1.61 ± 0.08c	1,649.79 ± 21.72d	1.24 ± 0.09b
G2	S1	136.67 ± 9.06a	1.61 ± 0.11a	1,197.19 ± 99.55a	1.09 ± 0.05a
	S2	148.00 ± 13.08a	1.42 ± 0.12a	1,302.00 ± 110.71ab	1.07 ± 0.06a
	S3	138.83 ± 14.75a	1.52 ± 0.14a	1,242.95 ± 52.83ab	0.98 ± 0.08a
	H1	174.17 ± 7.85a	2.13 ± 0.15b	1,643.00 ± 75.55c	1.09 ± 0.12a
	H2	161.00 ± 22.87a	2.01 ± 0.09b	1,843.76 ± 144.88d	1.09 ± 0.10a
	H3	160.33 ± 17.56a	2.04 ± 0.16b	2,072.57 ± 100.11e	1.05 ± 0.11a
	FWH1	149.40 ± 24.11a	2.18 ± 0.14b	1,272.48 ± 70.07ab	0.97 ± 0.06a
	FWH2	157.27 ± 10.78a	2.05 ± 0.09b	1,338.90 ± 64.53ab	1.06 ± 0.09a
	FWH3	147.67 ± 11.55a	2.01 ± 0.15b	1,392.05 ± 126.38b	1.02 ± 0.04a

G1 is the first cycle (3 March to 8 April 2021), G2 is the second cycle (9 April to 6 May 2021), S1 (0.7 *AE_p_*) S2 (0.9 *AE_p_*), and S3 (1.1 *AE_p_*); *AE_p_* is accumulated daily pan evaporation. H1 (0.7 HS), H2 (1 HS), and H3 (1.3 HS); HS is half-strength Hoagland solution. FWH1 (H1 switched to fresh water before harvest), FWH2 (H2 switched to fresh water before harvest), and FWH3 (H3 switched to fresh water before harvest). The lettuce quality characteristics were compared among different treatments in each growth cycle. Different letters indicate significant difference at *p* ≤ 0.05 tested with one-way ANOVA.

During G2, the ascorbic acid, soluble sugar content, and crude content were not influenced by nutrient solution concentration in HPS (**Table 1**). The nitrate content of lettuce in HPS (1.64, 1.84, and 2.07 g kg^−1^ under treatments H1, H2, and H3, respectively) was decreased by replacing the nutrient solution with fresh water prior to harvest to 1.27, 1.34, and 1.39 g kg^−1^ under treatments FWH1, FWH2, and FWH3, respectively. No additional nutritional quality parameters were influenced by fresh water replacement (**Table 1**).

### 3.8 Economic evaluation

#### 3.8.1 Initial investment

The initial investment of the two culture systems is illustrated in **Table 2**. Land and greenhouse construction costs were not included in the initial investment, since this study was conducted to compare the performances of the two typical culture systems under an identical greenhouse environment. The initial investment in HPS was $17,813, which was 21.76 times higher than that for SBS due to high cost of the growth channel ($8,805), frame ($3,807), and nutrient solution tank ($4,222).

**Table 2:** Initial investment for the two production systems.

Item	Description	Unit	Unit value ($)	Quantity	Initial cost ($)	Useful life years ($)	Annual cost ($)	Per growth cycle cost ($)
Soil-based system								
Automatic pan evaporation meter	Custom-made	−	130	1	130.43	10	13.04	1.63
Irrigation pump	15 m^3^ h^−1^	−	391.31	1	391.31	10	39.13	4.89
Irrigation pipe	*Φ*75 mm	m	1.74	150	260.87	10	26.09	3.26
Differential pressure tank	100 L	−	36.05	1	36.05	10	3.61	0.45
				Subtotal =	819		82	10
Hydroponic production system								
Growth channel	80 mm × 600 mm (Height × Width)	m	8.70	1012	8,804.69	10	880.47	88.05
Frame	Galvanized iron pipe	m	1.45	2631	3,806.56	10	380.66	38.07
Nutrient solution tank	Reinforced concrete tanks	m^3^	211.09	20	4,221.78	10	422.18	42.22
Installation	Labor	hour	3.52	40	140.60	10	14.06	5.07
Nutrient solution pump	25 m^3^ h^−1^	−	507.25	1	507.25	10	50.72	1.59
Portable pH meter	Shanghai Precision Instrument Company, China (HPB-4)	−	158.69	1	158.69	10	15.87	1.74
Portable EC meter	Shanghai Precision Instrument Company, China (DDB-303A)	−	173.91	1	173.91	10	17.39	
				Subtotal =	17,813		1781	176

#### 3.8.2 Operating cost

**Table 3** explains the operating costs of the two culture systems during each growth season. The total operating cost in HPS was $971, which was 47% higher than that of SBS ($660). In HPS, the sum costs of seed ($330) and growth media for germination ($124) in addition to fertilizer ($251) were more than 70% of the total operating costs. In SBS, labor cost ($261) was 1.3 times higher than that in HPS, accounting for 40% of the total operating cost.

**Table 3:** Operating costs of the two production systems.

Item	Description	Unit	Quantity	Unit value ($)	Total value ($)
Soil-based system					
Lettuce seed	*Lactuca sativa* L. cv. Flandria	Package	3.85	50.00	192.50
Germination growth media	Moss peat	Package	2.56	32.84	84.07
Land preparation	Labor	Hour	12.00	3.62	43.44
Transplanting	Labor	Hour	20.00	3.62	72.40
Organic fertilizer	Chicken manure	1,000 kg	0.60	59.70	35.82
Water-soluble compound fertilizer	N:P_2_O_5_:K_2_O (19:19:19)	1,000 kg	0.05	1,194.03	59.70
Irrigation and fertilizer application	Labor	Hour	5.00	3.62	18.10
Weed control	Labor	Hour	5.00	3.62	18.10
Electricity	Climate control	kW	375.00	0.07	26.25
Electricity	Irrigation pump	kW	12.00	0.07	0.84
Harvesting	Labor	Hour	30.00	3.62	108.60
				Subtotal =	660
Hydroponic production system					
Lettuce seed	*Lactuca sativa* L. cv. Flandria	Package	6.60	50.00	330.00
Germination growth media	Moss peat	Package	3.79	32.84	124.46
Transplanting	Labor	Hour	12.00	3.62	43.44
Hydroponic nutrient solution	Puleshou (hydroponic fertilizer)	1,000 kg	0.24	1,044.77	250.75
Disinfectant	hypochlorous acid (500 ml)	Bottle	2.00	1.00	2.00
Equipment operation	Labor	Hour	6.00	3.62	21.72
Electricity	Nutrient solution pump	kW	1,680.00	0.07	117.60
Electricity	Climate control	kW	375.00	0.07	26.25
Harvesting	Labor	Hour	15.00	3.62	54.30
				Subtotal =	970

#### 3.8.3 Economic parameters

The highest gross revenue, *NPV*, and net return (**Table 4**) were obtained under treatment H3 during G1 and H2 during G2. The highest *BCR* was found under the same treatment S3 during both G1 and G2, due to the lower initial investment of SBS. The gross revenue, *NPV*, and net return of treatment H1 were lower than treatments H2 and H3 under HPS, owing to the inhibition of lettuce growth due to the low nutrient solution concentration (**Figure 5**). In SBS, the influence of irrigation amount on the economic parameters during G1 was not due to high initial soil water content at the beginning of the experiment. Lower economic parameters were gained under the lowest irrigation level of S1 during G2.

**Table 4:** Economic parameters, gross revenue, net present value (*NPV*), benefit–cost ratio (*BCR*), and net return, for each treatment during the two growth cycles.

Production system treatment		Gross revenue cycle ($)		Net present value ($)		Benefit–cost ratio ($)		Net return cycle ($)	
		G1	G2	G1	G2	G1	G2	G1	G2
Soil-based system	S1	1,481.36	964.38	37,023.24	9,739.51	3.59	2.45	811.57	294.59
	S2	1,610.61	1,133.39	43,844.17	18,659.19	4.10	2.88	940.82	463.60
	S3	1,680.20	1,242.75	47,516.98	24,430.75	4.27	3.16	1,010.41	572.96
Hydroponic production system	H1	1,823.65	1,397.57	41,500.76	13,392.52	1.49	1.15	676.40	250.32
	H2	2,761.04	1,960.00	103,338.89	50,495.40	2.26	1.61	1,613.79	812.75
	H3	3,050.78	1,499.83	122,452.50	20,138.49	2.50	1.23	1,903.53	352.58

G1: the first cycle (3 March to 8 April 2021), G2: the second cycle (9 April to 6 May 2021). S1 (0.7 *AE_p_*) S2 (0.9 *AE_p_*), and S3 (1.1 *AE_p_*); *AE_p_* is accumulated daily pan evaporation. H1 (0.7 HS), H2 (1 HS), and H3 (1.3 HS); HS is half-strength Hoagland solution.

### 3.9 Multicriteria overall performance assessment

**Figure 11:**
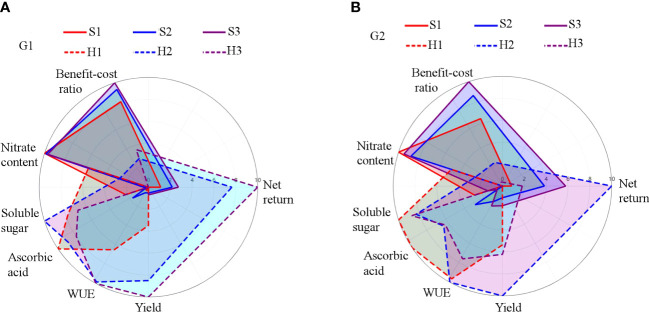
Multicriteria assessment of treatments. G1 **(A)** the first cycle (3 March to 8 April 2021), G2 **(B)** the second cycle (9 April to 6 May 2021). H1 (0.7 HS), H2 (1 HS), and H3 (1.3 HS); HS is half-strength Hoagland solution. S1 (0.7 *AE_p_*), S2 (0.9 *AE_p_*), and S3 (1.1 *AE_p_*); *AE_p_* is accumulated daily pan evaporation. The larger covered areas of radar map represent the better performance of lettuce production system for each treatment.

The HPS performed better regarding yield, *WP*, net revenue, and nutritional qualities (ascorbic acid and soluble sugar), but received lower scores for the indicators nitrate content and *BCR* (**Figure 11**). In contrast, SBS had better performance regarding nitrate content and *BCR* while the yield and nutritional qualities and net return were lower. Peak performance was obtained under treatments H3 (the covered area in the radar maps of 116.84) during G1 and H2 (the covered area in the radar maps of 115.89) during G2. For the average of two crop growth seasons, the highest composite performance (i.e., the average covered area in the radar maps of 110.90) was found for the H2 treatment. Moreover, the average covered area in the radar maps of 40.23, 49.46, 64.70, 53.33, and 76.34 was also found for the S1, S2, S3, H1, and H3 treatments, respectively.

## 4 Discussion

### 4.1 Crop growth

We know from widely published examples in the literature that the advantages of HPS include no external water or fertilizer discharge, enabling high yields, and prevention of soil-borne diseases (Lee and Lee, 2015; Magwaza et al., 2020; Majid et al., 2021; Verdoliva et al., 2021). However, few studies have compared commercial HPS with SBS, particularly while evaluating optimal agronomic practices (e.g., water and nitrogen supplies) for each. In our study, lettuce fresh weight was influenced significantly by nutrient solution concentration in HPS (*p* ≤ 0.05) and irrigation water amount in SBS (*p* ≤ 0.05). As the root system was submerged continuously in nutrient solution to avoid water stress in HPS (Li et al., 2018), significantly higher shoot fresh weight was observed for the treatment of highest yield (H3) compared with that of SBS (S3) during G1. However, there was no significant difference in shoot fresh weight between the two systems during G2, probably due to the hot weather and decreased dissolved oxygen solubility in the nutrient solution in SBS (Sharma et al., 2018).

Plant spacing has previously been shown to have a significant effect on plant-scale fresh weight and total yield per unit area (Van Quy et al., 2016). Plant spacing was reported by several researchers ranging from 30 to 50 cm between rows and 20 to 45 cm between lettuce plants in SBS (Abu-Rayyan et al., 2004; Alahi et al., 2014; Yordanova and Nikolov, 2017). Higher plant spacing was set in SBS to facilitate management including seedling transplanting and weed and pest control. Such higher plant densities are typical in HPS (Seo et al., 2009; Barbosa et al., 2015; Sace and Estigoy, 2015). Maboko and Du Plooy (2009) compared a range of hydroponic lettuce densities from 20 to 50 plant m^−2^ and found the highest yield with the closest spacing of 50 plant m^−2^. However, it should be noted that too high of a plant density will result in light competition due to dense canopy cover (Lam et al., 2019). Xu et al. (2020) also investigated the effects of photosynthetic photon flux density provided by LEDs and lighting strategies on the growth and tipburn occurrence in lettuce plants grown in HPS. The results show that lettuce shoot and root fresh weight and dry weight clearly increased with increases of light intensity and were not affected by different lighting strategies.

### 4.2 Environmental temperature

The environmental temperature is also a key factor affecting crop growth and yield and can be influential to *WP* (Gruber et al., 2011). Thompson et al. (1998) found that the optimum nutrient solution temperature for hydroponic lettuce was 24°C;. In our study, the maximum root zone temperature during G1 and G2 was 24.8 and 27.9°C; in HPS, and 19.4 and 21.9°C; in SBS, respectively, indicating that larger fluctuations in HPS correlated with diurnal fluctuations of atmospheric temperature in greenhouse (**Figure 3**). During G2, the higher nutrient solution temperatures would be expected to inhibit lettuce growth. Wurr et al. (1992) reported that lettuce is well adapted to a temperature range from 17 to 28°C; (day) under outdoor conditions. In our greenhouse, the average daytime temperatures were 15 to 26°C; and 18 to 29°C; during G1 and G2, respectively. The lettuce during G2 likely suffered from high-temperature stress, causing elongation (Yan et al., 2021). Therefore, the crop growth season during G2 was shortened to 28 days, compared with the growth season of 36 days during G1. Higher temperature additionally increases transpiration requirements without necessarily benefiting growth and yield (Sadok et al., 2021). Thakulla et al. (2021) also reported that water temperature affected root and shoot fresh and dry weight, plant width, and Brix for lettuce. Lettuce grown at 21.1°C were 15% greater for shoot fresh weight than plants grown at ambient conditions. In the current study, this was reflected by the more than 30% lower *WP* (**Figure 10**) due mostly to higher evapotranspiration (**Figure 6**) corresponding to higher temperatures during G2 compared to G1 (**Figure 3**). Both air and nutrient solution temperatures can be controlled and maintained within the optimal temperature range for lettuce growth, but this may not be economically feasible due to the cost of power for operating cooling/heating systems (Kawasaki and Yoneda, 2019).

### 4.3 Product quality

Findings from previous studies reporting the potentially high nutritional value of hydroponically grown lettuce (Majid et al., 2021; Verdoliva et al., 2021) were supported by the significantly higher values of ascorbic acid and soluble sugar content in HPS-grown compared to SBS-grown lettuce in the current study (**Table 1**). A possible drawback to HPSs is the potential threat to human health posed by high rates of accumulated nitrates (**Table 1**; Bian et al., 2016). Lei et al. (2018) reported a method to reduce the accumulated nitrate in hydroponic lettuce by applying selenium to nutrient solution, but high concentration of selenium in nutrient solution would cause toxicity to crop (Ruiz et al., 2007). Bian et al. (2016) found that the nitrate content of lettuce in HPS could be reduced by supplying continuous red and blue light (Red : Blue = 4:1) to promote the enzymatic activities of nitrate reductase, but at the expense of high energy input. Matysiak et al. (2022) also noted that the application of the lowest photosynthetic photon flux density of 160 µmol m^−2^ s^−1^ and 16 h photoperiod (9.2 mol m^−2^ per day of daily light integral) resulted in the lowest fresh weight, number of leaves, and head circumference of romaine lettuce in HPS. The level of nitrate was also below the limit imposed by European Community Regulation. Mozafar (1996) reported that the nitrate in vegetables was reduced by transferring the crop to nitrogen-free media prior to harvest. By eliminating 90% of N in nutrient solution 7 days before harvest, the nitrate content of endive (*Chicorium endivia* L. var. crispum Hegi) leaves was decreased by 42% (Martignon et al., 1994). This strategy to avoid high nitrate content in harvested lettuce in HPS was found to be successful as well in our study. Replacing the nutrient solution with pure fresh water 3 days before harvest reduced the nitrate content of HPS-grown lettuce compared to that found in SBS-grown lettuce (**Table 1**). Moreover, the contents of positive quality parameters, such as soluble sugar and ascorbic acid, were not influenced by the nutrient solution replacement.

### 4.4 Economic performance

The economic analysis parameters (i.e., cross revenue, *NPV*, and net return) during G2 were lower than that during G1, reflecting lettuce growth inhibition and lower economic yields due to higher air temperature in greenhouse during the second period. The benefit of greenhouse production was clearly influenced by meteorological environmental conditions, specifically air temperature. The total gross revenue in HPS was higher than that in SBS, due to the higher total yield of lettuce. The *BCRs* of the two culture systems were both higher than 1, indicating that both of the systems could be lucrative (Grafiadellis et al., 2000). The highest net return was obtained under hydroponic treatments, indicating that the HPS was more profitable compared with SBS. Majid et al. (2021) also reported that the HPS performed better than SBS on economic analysis parameters. In China, where irrigation decision-making is commonly based on traditional knowledge or experience, the initial investment of SBS (**Table 2**) could therefore be negligible and worthwhile considering the lifespan and potential profits of a typical commercial sized greenhouse.

There was obvious difference in net return among the three nutrient concentration treatments in HPS, indicating that successful hydroponic production requires both skilled and accurate management of technologies regarding control and adjustments of both nutrient solution and growing environment.

### 4.5 Multicriteria performance

With the deterioration of the soil quality in greenhouses under traditional soil cultivation, a rising demand for high-quality vegetables, and shortage of farmland and water resources, it is increasingly urgent to develop advanced methods for assuring high yields of good-quality agricultural products while affording farmers motivation via maximum economic return. Our results showed that the HPS was advantageous regarding *WP*, yield, net return, and nutritional quality parameters (**Table 1**; **Figure 10**) compared to SBS. The problematic issue of potentially high lettuce nitrate content under HPS was found to be resolved by replacing the nutrient solution with fresh water 3 days before harvest (**Table 1**). Higher initial investment led to lower *BCR* score under HPS, which is one of the biggest barriers for expanding its adoption expansion (Zhang et al., 2018; Chowdhury et al., 2020). However, the high initial investment of HPS can be overcome by higher economic return. The difference in multicriteria performance under the same treatment of HPS between G1 and G2 indicated that the production of lettuce under HPS was influenced greatly by the environmental conditions due to the poor temperature buffering capacity of the nutrient solution. The difference in multicriteria performance among the three treatments under HPS in the same growth season showed that the lettuce production was affected by the agronomic measures of nutrient solution management. Therefore, advanced technologies for smart and precise management of nutrient solution and environmental control in greenhouse are expected to benefit and enable highly profitable vegetable production under HPS (Gruda, 2008).

### 4.6 Environmental implications

Conventional soil-based vegetable production systems tend to have wide negative impact to local environments including indiscriminate use of chemicals and pesticides, soil salinization, soil acidification, and nitrate accumulation, which together represent potential contamination risk of groundwater (Shi et al., 2009; Han et al., 2015; Sharma et al., 2018). HPS is considered a clean, safe, and environment-friendly vegetable production technique because it eliminates risks of soil-borne disease and minimizes insect or pest infection to the crops, thereby reducing use of pesticides and their resulting toxicity (Khan et al., 2021). Under closed hydroponics, the waste nutrient solution can be renewed for crop cultivation through filtering, sterilizing, and adjusting the nutrient solution (Sharma et al., 2018). The leaching of drain water from the root zone into deep soil is prohibited to prevent environmental pollution. Hydroponics can be regarded as part of ecological systems designed to improve the environment by increasing humidity and lowering temperature in arid climates (Schnitzler, 2013). Fresh vegetables can be supplied to consumers locally with near-zero net carbon emissions from HPS operations due to minimal transportation needs (Schnitzler, 2013). However, Blom et al. (2022) evaluated the current carbon footprint of lettuce produced in a vertical farm (VF) in comparison to conventional open-field farming (OF) and both SBS and HPS in Netherlands. The results showed that the carbon footprint of the VF is 8.177 kgCO_2-eq_ kg^−1^, 16.7 times greater than that of the OF, 6.8 times greater than SBS, and 5.6 times greater than HPS. The results also implied that the annual yields of HPS are 1.8 times greater than SBS, and the carbon emission of HPS is 1.2 times greater than SBS. This may be related to the fact that HPS consumes more electricity and fuel than SBS.

## 5 Conclusion

Increasing and ensuring local fresh vegetable supply is critical for the sustainable development of large cities. The present study systematically analyzed lettuce production under two typical culture systems, the first being soil-based (SBS) and the second being hydroponic (HPS). Compared with SBS, HPS obtained higher yield, quality, and *WP*, signifying its potential to reduce the demand for farmland and water resources in vegetable production. The accumulated nitrate in HPS was decreased by replacing the nutrient solution with fresh water, with no influence on the other nutritional quality parameters.

Commercial HPS was found to be more profitable compared with SBS under optimal climate conditions. However, higher initial investment was required to construct the modern commercial hydroponic leafy vegetable production system with high returns. Notice that the HPS is more sensitive to the air temperature and agronomic management measures, due to the poor temperature buffering capacity of nutrient solution.

In general, we conclude that HPS has superior competitiveness for urban leafy vegetable production compared with SBS as long as the necessary technologies and management for controlling and optimizing greenhouse and nutrient solution for high-yield-quality vegetable production can be provided and practiced. We believe that in the near future, automatic equipment and protocols for environment control and fertilizer regulation should be developed to reduce the dependence on manual operation.

## Data availability statement

The raw data supporting the conclusions of this article will be made available by the authors, without undue reservation.

## Author contributions

LW: Conceptualization, Methodology, Investigation, Formal analysis, Software, Writing – original draft, Writing – review and editing. SN: Conceptualization, Methodology, Visualization, Software, Writing – original draft, Writing – review and editing. WZ: Formal analysis, Funding acquisition. JG: Methodology, Investigation. YLL: Formal analysis, Investigation. YKL: Methodology, Investigation. XC: Data curation, Software. AB-G: Writing – review and editing. XW: Funding acquisition, Data curation. All authors contributed to the article and approved the submitted version.
